# Achieving Low Dielectric Loss and High Humidity Stability Polyimide Through the Synergistic Effect of Copolymer Monomer Optimization and Aggregation State Regulation

**DOI:** 10.3390/polym18131595

**Published:** 2026-06-26

**Authors:** Xing Kang, Chenyu Liu, Hongkui Wu, Runxin Bei, Siwei Liu, Yi Zhang

**Affiliations:** 1 PCFM Lab, GD HPPC Lab, Guangdong Engineering Technology Research Center for High-Performance Organic and Polymer Photoelectric Functional Films, GBRCE for Functional Molecular Engineering, State Key Laboratory of Optoelectronic Materials and Technologies, School of Chemistry, IGCME, Sun Yat-sen University, Guangzhou 510275, China; kangx7@mail2.sysu.edu.cn (X.K.); beirunxin1@shunxnm.cn (R.B.); ceszy@mail.sysu.edu.cn (Y.Z.); 2 Wuxi Shunxuan Photoelectric Technology Co., Ltd., Yixing 214242, China; 3 National Engineering Research Center of Electronic Circuits Base Materials, SHENGYI Technology Co., Ltd., Dongguan 523808, China; wuhk@syst.com.cn

**Keywords:** polyimide, aggregation state, water adsorption, high-frequency dielectric properties

## Abstract

Polyimide (PI) has excellent comprehensive performance and a wide range of applications, but its high molecular chain rigidity, poor flexibility, and difficulty in processing and molding limit its further applications. Introducing flexible groups, fluorinated groups, and copolymer modification are effective methods to improve PI performance. Among them, ether bonds can enhance the mobility of molecular chains and optimize processing performance, trifluoromethyl groups can improve material crystallinity and high-frequency dielectric properties, and copolymer modification can achieve the regulation of aggregated structures. In this study, the rigid TFMB-BPDA polyimide system was used as the matrix, and various ether-bond-containing diamine monomers were introduced through copolymerization, to explore the effects of different types of ether-bond-containing diamines and the soft-to-hard segment ratios on the aggregated structure and high-frequency dielectric properties of PI films. The goal is to optimize the comprehensive performance of PI materials through molecular structure design.

## 1. Introduction

In recent years, polyimide (PI) has been widely used in aerospace, electronic components, and high-performance film materials due to its excellent thermal stability, electrical insulation, and mechanical strength [1,2,3]. Although PI demonstrates outstanding performance in these applications, its high rigidity results in poor flexibility and, during processing, it faces challenges such as difficulty in forming and complex processing steps. These drawbacks significantly limit the expansion of PI applications in specific scenarios where material flexibility and ease of processing are highly required. To address this issue, researchers have adjusted the structure of PI by introducing flexible groups, thereby optimizing its performance [4,5,6,7,8,9,10,11,12,13].

Introducing some flexible groups, such as ether bonds, into polyimides can effectively improve the processability of the material [10]. As a flexible group, ether bond allows the PI molecular chains to bend to a certain extent, thereby reducing interactions between the chains, increasing chain mobility, and altering the aggregated structure of the PI. In addition, the low polarity of ether bonds helps decrease intermolecular forces. Therefore, PIs containing ether bonds have significant advantages in adjusting the aggregated structure, mechanical properties, and thermal stability of polyimides. On the other hand, some PIs containing trifluoromethyl groups exhibit crystallinity. For example, Mitsui company [14] introduced hexafluoroisopropyl-substituted biphenyl units into the Aurum diamine structure and successfully synthesized new fluorinated polyimide materials. The introduction of fluorinated groups not only significantly enhances the crystallinity of polyimides but also optimizes their dielectric properties, especially demonstrating excellent electrical performance in high-frequency environments. During the polyimide polymerization process, copolymerization can be carried out by introducing a third monomer, which allows for the regulation of the aggregated structure within a reasonable monomer ratio range. Related research focuses on selecting suitable comonomers and proportion parameters, aiming to further optimize the material’s thermal stability and melt processability while maintaining its original excellent properties.

Based on the above background, we introduced diamines with different ether linkages into the TFMB-BPDA (2,2′-bis(trifluoromethyl)benzidine and 3,3′,4,4′-biphenyltetracarboxylic di-anhydride) system to regulate its aggregated structure. The TFMB-BPDA system is a polyimide system with a rigid biphenyl structure as the main chain, offering good thermal stability and mechanical properties. By incorporating soft-segment diamines containing ether linkages, the flexibility and rigidity of the polymer can be adjusted, thereby controlling its aggregated structure. We systematically investigated the effects of different soft-segment diamines and their ratios with rigid chain segments on the aggregated structure and high-frequency dielectric properties of PI films.

## 2. Materials and Methods

### 2.1. Materials

TFMB (2,2′-bis(trifluoromethyl)benzidine), BPDA (3,3′,4,4′-biphenyltetracarboxylic di-anhydride), BAPB (4,4′-bis(4-aminophenoxy)biphenyl) and aODA (3,4′-oxydianiline) were purchased from China Tech (Tianjin) Chemical Co., Ltd. (Tianjin, China) ODA (4,4′-oxydianiline) was purchased from Jiangsu Aikon (Nanjing, China). Except for TFMB, which was sublimated before use, all other monomers were dried in a vacuum oven prior to use. N-Methyl-2-pyrrolidinone (NMP, Extra Dry) was purchased from Energy Chemical (https://www.energy-chemical.com/) and used as received.

### 2.2. Preparation and Characterization of the PI Films

**Figure 1:**
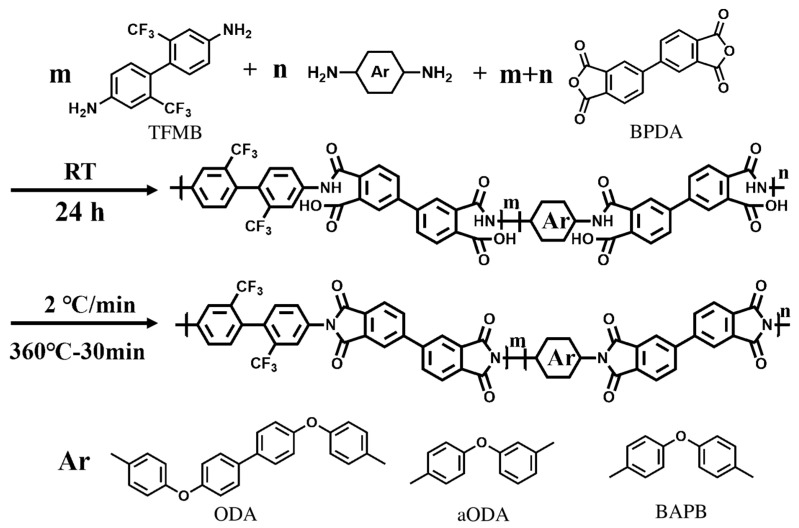
Schematic diagram of the synthesis steps of copolymer polyimide.

Polyimide films were synthesized via a two-step method. The specific steps are illustrated using the TFMB-BPDA as an example. Step 1, synthesis of polyamic acid (PAA): In a dry glass sample bottle, TFMB (1.6012 g, 5.0 mmol) was added to NMP (15 mL) and stirred at room temperature until fully dissolved. Then BPDA (1.4710 g, 5.0 mmol) was added, and the mixture was continuously stirred at room temperature for 24 h to obtain a viscous PAA solution. Step 2, thermal imidization: The obtained PAA solution was degassed in a vacuum oven and then uniformly coated onto a clean glass plate. It was pre-baked on a hot plate at 100 °C for 30 min, then transferred to a nitrogen-atmosphere oven and heated at a rate of 2 °C per minute up to 360 °C. The temperature was maintained at 360 °C for 30 min to obtain the TFMB-BPDA polyimide film. The polyimides with different feed ratios and their synthetic routes are shown in Figure 1 and Table S1.

After thermal imidization, we obtained thirteen PI films with different compositions. In the infrared spectra (Figure S1), all these PI films exhibit characteristic absorption peaks of the imide ring, including the C=O stretching vibration peaks between 1700–1750 cm^−1^ and 1770–1800 cm^−1^, as well as the C-N stretching vibration peaks in the range of 1300–1400 cm^−1^. These characteristic peaks confirm that the molecular structure of the PI films meet expectations.

## 3. Results

### 3.1. Comparative Analysis of Dielectric Loss Factors of the Obtained Copolymer and Homopolymer PI Films

By comparing the dielectric loss factors (*D*_f_) of different copolymer monomers with the BPDA homopolymer, the results show that the *D*_f_ values of all copolymer PI films are lower than those of the corresponding homopolymers. At 50% relative humidity, the *D*_f_ value of TFMB-BPDA is 4.75 × 10^−3^, while that of BAPB homopolymer is 4.25 × 10^−3^ (Table S2). In comparison, the *D*_f_ values of BAPB and TFMB copolymer PI films range from 3.08 × 10^−3^ to 3.56 × 10^−3^, which are significantly lower than those of the homopolymers TFMB-BPDA and BAPB-BPDA. Similarly, the *D*_f_ value of ODA homopolymer is 4.25 × 10^−3^, whereas the *D*_f_ values of ODA copolymers range from 3.25 × 10^−3^ to 3.94 × 10^−3^. For aODA, the homopolymer has a *D*_f_ as high as 6.47 × 10^−3^, while the *D*_f_ values of the aODA copolymer systems decrease to 2.80 × 10^−3^~3.97 × 10^−3^, all lower than those of aODA-BPDA and TFMB-BPDA. The results show that the *D*_f_ of all synthesized copolymers of the three different monomers is lower than that of their corresponding homopolymers.

### 3.2. Effect of Copolymer Ratio on the Df of PI Films

For the TFMB/BAPB copolymers, as the proportion of BAPB increases, the *D*_f_ value first decreases and then rises (Table S2). Under 50% relative humidity, the *D*_f_ value of the homopolymer BPDA-BAPB is 4.25 × 10^−3^, whereas when the BAPB content is 15%, *D*_f_ significantly decreases to 3.08 × 10^−3^, a reduction of 27.5%. However, as the BAPB proportion continues to increase to 50% (50TFMB/50BAPB), the *D*_f_ value rises again to 3.56 × 10^−3^, though it remains 16.2% lower than that of pure BAPB homopolymer. For the TFMB/ODA copolymers, the copolymer ratio has a more significant effect on *D*_f_. In the case of 50% copolymerization (50TFMB/50ODA), the *D*_f_ value decreases to 3.28 × 10^−3^, a reduction of 22.8%. When the ODA proportion increases from 15% to 50%, the *D*_f_ value gradually decreases from 3.94 × 10^−3^ to 3.28 × 10^−3^, with no obvious rebound observed, and the change in *D*_f_ is more gradual. In contrast, the copolymerization effect of aODA exhibits a more complex trend. The *D*_f_ of aODA homopolymer is 6.47 × 10^−3^, but at a 50% copolymerization ratio, the *D*_f_ value significantly decreases to 3.10 × 10^−3^, a reduction of 52.1%. As the proportion of aODA increases, *D*_f_ shows a trend of first decreasing and then rising, specifically: 85TFMB/15aODA (3.97 × 10^−3^), 70TFMB/30aODA (2.80 × 10^−3^) and 50TFMB/50aODA (3.10 × 10^−3^). Among the prepared PI films, the PI film with a 30% aODA copolymer ratio has the lowest *D*_f_ value of 2.8 × 10^−3^.

### 3.3. The Effect of Diamine Monomers at the Same Copolymerization Ratio

Under the same copolymerization ratio, different diamine monomers show significant differences in regulating *D*_f_. Taking the data at 50% humidity as an example, at a 15% copolymerization ratio, the *D*_f_ values of the different monomer systems are ranked as follows: BAPB (3.08 × 10^−3^) < ODA (3.94 × 10^−3^) < aODA (3.97 × 10^−3^). When the copolymerization ratio is increased to 30%, the aODA system shows a significant advantage, with a *D*_f_ value (2.80 × 10^−3^) that is 15.9% and 13.8% lower than that of BAPB (3.33 × 10^−3^) and ODA (3.25 × 10^−3^), respectively. At a 50% copolymerization ratio, the *D*_f_ values of TFMB/50BAPB, TFMB/50ODA, and TFMB/50aODA are 3.56 × 10^−3^, 3.28 × 10^−3^, and 3.10 × 10^−3^, respectively, representing decreases of 16.2%, 22.8%, and 52.1% compared to their respective homopolymers.

By regulating the type and proportion of copolymer monomers, we achieved optimization of the dielectric loss factor (*D*_f_) of polyimides. Notably, at a 30% copolymer ratio, the aODA copolymer system exhibited the best performance (*D*_f_ = 2.80 × 10^−3^, 50% RH), with its dielectric loss reduced by 52.1% compared to pure aODA homopolymer; in contrast, the ODA copolymer system showed relatively mild changes in dielectric loss. The results indicate that selecting copolymer monomers with moderate flexibility is an effective strategy for developing low-*D*_f_ polyimide materials.

### 3.4. The Influence of the Aggregated Structure of Thin Films on Material Properties

**Figure 2:**
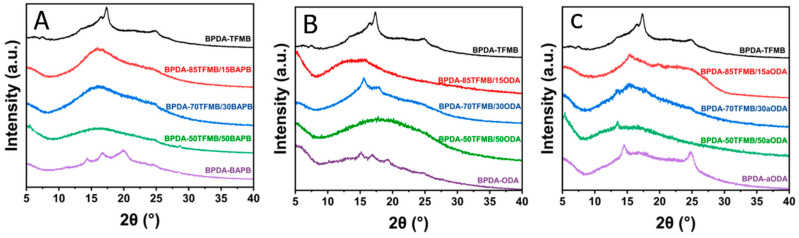
Wide-angle X-ray diffraction of the obtained homopolymer and copolymer PI films: (**A**) BAPB polymers; (**B**) ODA polymers; (**C**) aODA polymers.

We studied the changes in the aggregated structure of the BPDA-TFMB system after introducing different diamine comonomers through WAXD (Figure 2). The presence of rigid and linear chain structures usually leads to denser molecular packing, thereby increasing the likelihood of crystallization. The homopolymer system of BPDA-TFMB has a certain degree of crystallinity, corresponding to sharp and high-intensity diffraction peaks. As the content of BAPB gradually increases, the diffraction peak positions shift, revealing changes in interplanar spacing and the disruption of the crystal structure. At the same time, the peak intensity decreases and the peak shape broadens, indicating that BAPB disrupts the originally orderly crystal arrangement of BPDA-TFMB, resulting in a decrease in crystallinity and a transition of the aggregated structure toward disorder. The introduction of ODA brings similar changes. The fluctuations in diffraction peak positions reflect alterations in molecular chain arrangement and interplanar spacing, while the significant weakening of peak intensity and broadening of peaks further indicate that copolymerization with ODA greatly reduces the material’s crystallinity, promoting its development toward an amorphous state. The addition of aODA has the same effect in disrupting the crystal structure, weakening material order, and deepening the degree of amorphization.

In addition, the -CF_3_ group slightly increases the side chain volume, which may interfere with the close packing of adjacent molecular chains [15]. In BPDA-TFMB, the regularity of the rigid biphenyl main chain dominates the crystallization ability, and the symmetrically substituted -CF_3_ only causes limited interference. However, the introduction of soft segments disrupts the continuity of the rigid main chain structure and increases free volume; its steric hindrance and polarity effects are amplified, thereby reducing the degree of crystallinity [16].

After introducing soft segment diamine copolymerization into the BPDA-TFMB system, the evolution of the aggregated structure is significantly synergistically correlated with the reduction in dielectric loss (*D*_f_). Its mechanism can be analyzed from the multi-scale coupling of molecular structure, different micro-area characteristics, and polarization behavior [17]. The soft segments disrupt the regular packing of the original rigid aromatic segments in BPDA-TFMB, leading to a decrease in the system’s crystallinity. The soft segments (low polarity) and hard segments (rigid aromatic chains) form a different micro-area characteristics structure, coexisting regions enriched in hard segments (short-range order or locally dense packing) and regions enriched in soft segments (completely amorphous). This two-phase structure causes a differentiated distribution of free volume (FFV): in the hard segment regions, the rigidity of the molecular chains forms locally dense packing, significantly reducing the FFV and effectively limiting the vibration and rotational freedom of high-polarity groups (such as -CF_3_ and -C=O), thereby suppressing their relaxation losses under high-frequency electric fields; in contrast, although the soft segment regions have higher FFV, their low-polarity chemical structure (such as ether bonds and aliphatic chains) results in an extremely low dipole moment density (μ/V_vdw_), meaning that even with increased chain mobility, their polarization response to an external electric field remains negligible, avoiding potential losses caused by increased free volume. From the perspective of dielectric loss mechanisms, the low polarity of the soft segments reduces the overall μ/V_vdw_ through a polar dilution effect, weakening the coupling strength between dipoles and the external electric field [18]. The hard segment regions formed by different micro-area characteristics, which have selectively low FFV, induce spatial confinement on the motion of high-polarity groups through local dense packing, thereby reducing polarization hysteresis loss. In addition, the increased complexity of molecular chain conformations at the soft-hard segment interface forms kinetic barriers, further hindering the cooperative effects of dipole motion and reducing the polarization relaxation intensity. The high-frequency motion of the soft segment chains (such as ether bond vibrations) mismatches with the GHz-level electric field frequency, also leading to a decrease in energy dissipation efficiency. Therefore, the reduction in dielectric loss after the introduction of soft segments is essentially the result of the synergy between chemical structure regulation of dipole density and optimization of dipole motion through aggregated state structures. Specifically, this is achieved by using different micro-area characteristics to create locally low free volume (FFV) in the hard segment regions to restrict the movement of high-loss polar groups, while leveraging the low μ/V_vdw_ characteristics of the soft segment regions to avoid the negative effects of increased free volume. Ultimately, in a system dominated by amorphous aggregates, effective suppression of dielectric loss at high frequencies could be realized through the cooperation of multiscale structures.

Table 1 presents the relationship between *D*_f_ and *D*_k_ of TFMB-BPDA system copolymer PI films and relative humidity under different humidity conditions. The high-frequency dielectric properties of the PI films were measured using a P5003A network analyzer (Keysight Technologies, Santa Rosa, CA, USA) with a 10 GHz cavity resonator (QWED, Warsaw, Poland). Through curve fitting, the slope, intercept, and R^2^ value for each system were obtained, revealing the response patterns of different PI films to humidity changes. The *D*_f_ values of all PI films exhibit a strong linear relationship with humidity, and the R^2^ values of the fitted curves are all greater than 0.98, indicating a significant linear correlation between humidity and *D*_f_ values. Different copolymer systems exhibit their own response patterns to humidity changes. Although the *D*_f_ values of all copolymer systems increase gradually with increasing humidity, the magnitude of the increase is significantly influenced by the type and proportion of copolymer monomers. Different comonomers and their ratios have a significant impact on the humidity responsiveness of PI films. The BAPB system shows low humidity dependence, while the ODA system exhibits strong humidity sensitivity. The aODA system shows a notable suppression of humidity responsiveness after increasing the proportion of TFMB. These results indicate that adjusting the type and ratio of comonomers, especially selecting appropriate monomers, can effectively regulate the humidity stability and dielectric properties of PI films.

### 3.5. Optical Properties of the Prepared PI Films

**Figure 3:**
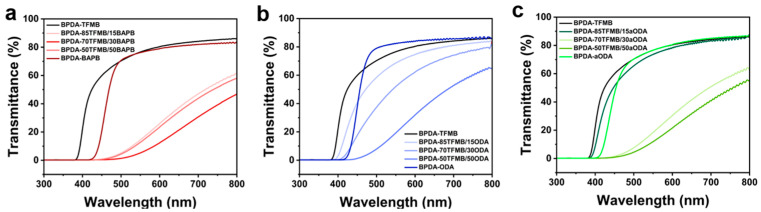
UV-visible spectra of PI films: (**a**) BAPB polymers; (**b**) ODA polymers; (**c**) aODA polymers.

Figure 3 shows the ultraviolet-visible (UV-Vis) transmittance of three different monomer copolyimide (PI) films as a function of wavelength. The figure is divided into three parts: (a) presents the BAPB copolymer system, (b) is the ODA copolymer system, and (c) is the aODA copolymer system. In Figure 3a, compared with BAPB/TFMB copolymer PI films, BPDA-TFMB and BPDA-BAPB maintain higher transmittance at longer wavelengths, while the transmittance of the copolymer systems decreases significantly. In Figure 3b, ODA copolymer PI films exhibit relatively high transmittance. The transmittance of all ODA copolymer systems is more uniform than that of the BAPB system and remains relatively stable with increasing wavelength. Notably, the transparency of BPDA-50TFMB/50ODA is significantly lower than that of other copolymer films. Figure 3c shows the transmittance of copolymer PI films containing aODA. In the aODA system, the trend of transmittance variation is similar to that of the ODA system, but the transmittance in the ultraviolet region is relatively lower. Specifically, the transmittance of BPDA-50TFMB/50aODA and BPDA-70TFMB/30aODA is significantly lower than that of other aODA copolymer systems, indicating that the transparency of PI films in these two systems is lower.

**Figure 4:**
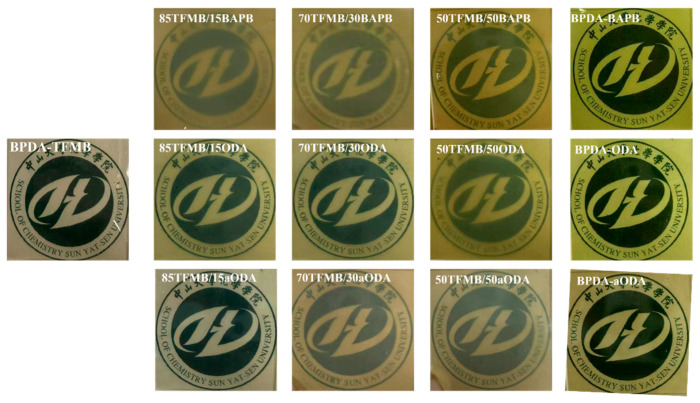
Optical photos of the 13 prepared BPDA-based PI films.

Figure 4 shows photos of all 13 types of PI films and also highlights the changes in transparency. The transparency of copolymer PI films is noticeably lower than that of homopolymer PI films. The disorder in the soft segments may cause differences in the arrangement and interaction of molecular chains between the soft and hard segments: when soft segments are introduced into the copolymer PI, their disordered nature may make them less compatible with the hard segments, easily leading to a mismatch with the wavelength of light and causing light scattering. Although polyimide as a whole may be amorphous, the disordered arrangement of the soft segments and the resulting differences in the arrangement with the hard segments enhance light scattering, which in turn makes the transparency of copolymer PI films significantly lower than that of homopolymer PI films. This series of phenomena strongly suggests that there are indeed microdomain arrangements with different characteristics in copolymer PI.

### 3.6. Thermodynamic Properties of the Prepared PI Films

**Figure 5:**
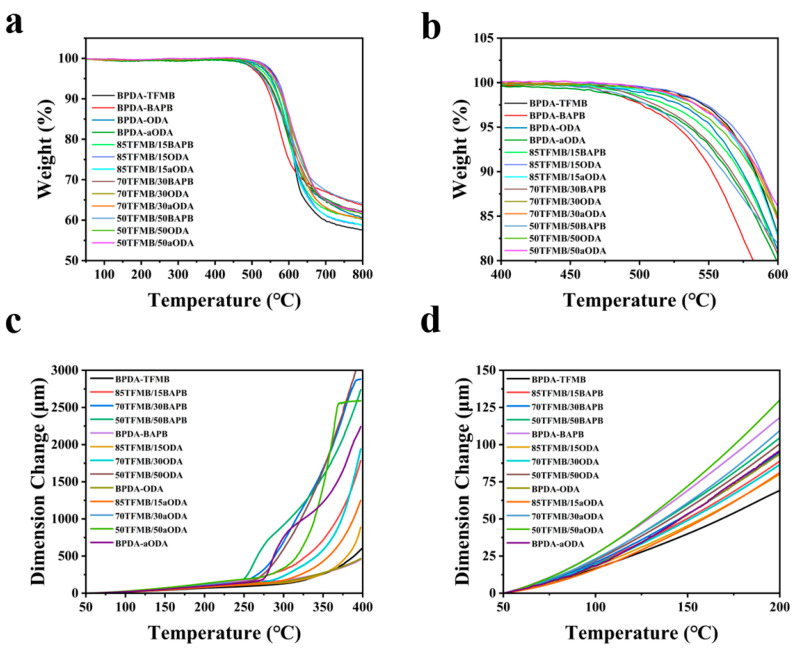
TGA and TMA of co-polyimide films: (**a**) TGA: 50–800 °C; (**b**) TGA: 400–600 °C; (**c**) TMA: 50–400 °C; (**d**) TMA: 50–200 °C.

Figure 5a,b show the thermal properties of the 13 types of PI films, with specific data provided in Table S3. The thermal decomposition temperature (*T*_d_) of BPDA copolymer PI films significantly depends on the chemical structure of the diamine monomer and the copolymer ratio. The homopolymer PI film of BPDA-TFMB exhibits excellent thermal stability, with initial decomposition temperature *T*_d_ 1 wt% and *T*_d_ 5 wt% reaching 522 °C and 563 °C, respectively, attributed to its rigid aromatic backbone and the electron effect of the trifluoromethyl group enhancing intermolecular interactions. However, as flexible or isomeric diamines are introduced through copolymerization, thermal stability shows a regular decline. Taking the TFMB/BAPB system as an example, when the BAPB ratio increases from 15% to 50%, the *T*_d_ 1 wt% decreases from 487 °C to 477 °C, and the *T*_d_ 5 wt% decreases from 545 °C to 530 °C, indicating that the introduction of BAPB reduces thermal stability. Similarly, in the TFMB/ODA copolymer system, as the ODA proportion increases, the *T*_d_ 1 wt% and *T*_d_ 5 wt% decrease from 522 °C/566 °C (15% ODA) to 509 °C/555 °C (50% ODA). It is noteworthy that the introduction of the isomeric diamine aODA has an even more significant impact on thermal stability: the *T*_d_ 1 wt% (467 °C) and *T*_d_ 5 wt% (534 °C) of pure aODA homopolymer are both lower than those of its isomer ODA (494 °C/551 °C), possibly due to the meta-substituted structure disrupting molecular symmetry. Additionally, in copolymer systems (such as 70TFMB/30aODA), the *T*_d_ 5 wt% stabilizes at 561 °C, suggesting that partial copolymerization may compensate for structural defects by restricting segmental motion.

Figure 5c,d show the CTE data of PI films prepared by copolymerizing different diamine monomers with BPDA. It can be observed that the CTE value is related to the structure of the diamine monomer and the copolymerization ratio. The PI film of TFMB homopolymer, which is highly rigid and contains fluorine, exhibits the lowest CTE (29.3 ppm·K^−1^), attributed to the rigid backbone of its molecular chain and the fluorine atoms restraining thermally induced chain segment motion. However, with the introduction of flexible diamine monomers through copolymerization, the CTE shows a gradual increasing trend. In the TFMB/BAPB copolymer system, as the proportion of the flexible diamine BAPB increases from 15% to 50%, the CTE rises from 37.1 ppm·K^−1^ to 44.5 ppm·K^−1^. When homopolymerized by BAPB, the CTE further increases to 50.9 ppm·K^−1^, indicating that flexible segments accelerate thermal expansion behavior by increasing molecular chain mobility and free volume. Similar trends are also observed in the TFMB/ODA and TFMB/aODA systems: when ODA is copolymerized with TFMB, the CTE increases from 33.8 ppm·K^−1^ (15% ODA) to 42.3 ppm·K^−1^ (50% ODA). Notably, the introduction of the isomeric diamine aODA has an even more significant effect on the CTE. When the aODA content reaches 30%, the CTE suddenly rises to 45.9 ppm·K^−1^, even higher than the 41.0 ppm·K^−1^ of pure aODA homopolymer. This may be due to its isomeric structure disrupting the symmetry and packing order of the molecular chains, further weakening interchain interactions.

**Figure 6:**
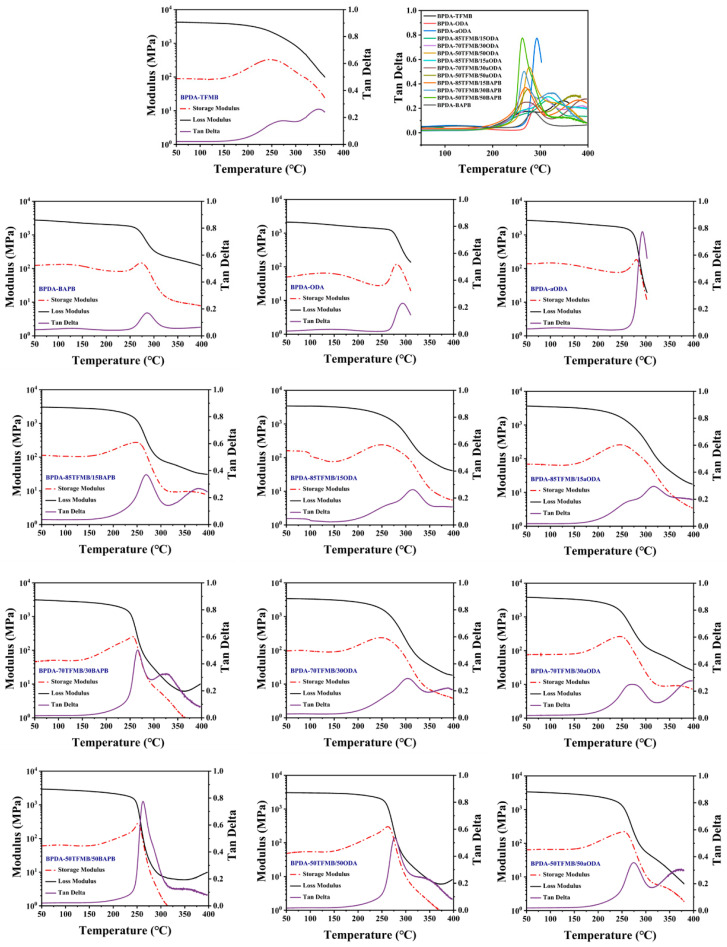
DMA test curves of the 13 prepared polyimide films.

Figure 6 shows DMA curves of the 13 types of PI films. The study results indicate that the glass transition temperature (*T*_g_) of PI films synthesized by copolymerizing BPDA with different diamine monomers exhibits significant composition dependence. When the rigid diamine TFMB is the main component, the Tg of its homopolymer reaches as high as 350 °C, attributed to the rigidity of the molecular chain enhanced by the fluorine substituents. However, with the copolymerization introduction of flexible or isomeric diamines (BAPB, ODA, or aODA), Tg shows complex variations. In the TFMB/BAPB copolymer system, the 85TFMB/15BAPB sample exhibits a double Tg (268 °C and 377 °C), suggesting possible different micro-area characteristics due to structural differences between the two diamines, corresponding to the TFMB-rich phase and BAPB-rich phase transitions, respectively. When the BAPB content increases to 50% (50TFMB/50BAPB), the Tg decreases to 262 °C, with only a single transition observed, implying improved chain segment compatibility and the formation of a homogeneous structure. Similar phenomena are found in the TFMB/ODA and TFMB/aODA systems; for example, the 70TFMB/30aODA shows a double *T*_g_ (272 °C and 394 °C), further confirming phase separation behavior, while copolymers with high contents of ODA or aODA significantly reduce the overall Tg (50TFMB/50ODA has a *T*_g_ of 277 °C). Notably, compared with ODA, the introduction of the isomeric diamine aODA leads to a higher degree of phase separation (such as the high *T*_g_ of 394 °C for 70TFMB/30aODA). In conclusion, by adjusting the type and ratio of diamines, the segmental mobility and phase structure of BPDA-based PI films can be effectively controlled.

## 4. Conclusions

This study systematically investigates the high-frequency dielectric properties of BPDA- and TFMB-based polyimide films under different humidity conditions, focusing on the regulation of *D*_f_ and humidity responsiveness using different copolymer monomers (such as BAPB, ODA, aODA) and their ratios. By introducing different soft-segment diamine monomers (BAPB, ODA, aODA) for copolymerization, the aggregated structure of the BPDA-TFMB system undergoes significant changes. The regularity of the rigid aromatic main chain is disrupted, crystallinity decreases with increasing copolymer ratio, diffraction peak intensity weakens, and peak shapes broaden, indicating a transition of the material toward an amorphous state. The flexible chain segments form different micro-area characteristic structures with rigid hard segments. Among them, copolymerization with BAPB and aODA causes a more pronounced disruption of crystallinity, while the ODA system shows a more gradual variation in crystallinity. Different copolymer monomers exhibit significant differences in regulating humidity sensitivity, and the system’s response to humidity can be adjusted through copolymerization. The synergistic effect of different micro-area characteristics and amorphization suppresses high-frequency dielectric loss through multi-scale mechanisms. Thereby, by chemically designing the copolymer monomers and regulating the aggregate structure, a balance of low dielectric loss and high humidity stability can be achieved in systems dominated by the amorphous state, providing an important theoretical basis for the development of high-performance polyimides.

## Figures and Tables

**Table 1:** Linear fitting of dielectric constant (*D*_k_) and dielectric loss (*D*_f_) with relative humidity.

Polyimide	*D*_f_ (Y) - RH (X): Y = a_1_X + b_1_			*D*_k_ (Y) - RH (X): Y = a_2_X + b_2_		
	a_1_	b_1_	R^2^	a_2_	b_2_	R^2^
BPDA-TFMB	0.0070	0.0012	0.99	0.37	2.94	0.97
BPDA-85TFMB/15ODA	0.0073	0.0006	0.99	0.46	2.93	0.95
BPDA-70TFMB/30ODA	0.0058	0.0007	0.98	0.19	3.14	0.97
BPDA-50TFMB/50ODA	0.0068	0.0002	0.99	0.18	3.16	0.92
BPDA-ODA	0.0104	0.0004	0.98	0.24	3.17	0.98
BPDA-85TFMB/15aODA	0.0060	0.0010	0.99	0.18	3.00	0.94
BPDA-70TFMB/30aODA	0.0037	0.0010	0.99	0.16	3.04	0.94
BPDA-50TFMB/50aODA	0.0043	0.0009	0.99	0.20	3.07	0.91
BPDA-aODA	0.0118	0.0007	0.99	0.20	3.14	0.91
BPDA-85TFMB/15BAPB	0.0039	0.0012	0.99	0.24	3.11	0.90
BPDA-70TFMB/30BAPB	0.0041	0.0013	0.99	0.22	3.09	0.93
BPDA-50TFMB/50BAPB	0.0042	0.0015	0.99	0.13	3.07	0.90
BPDA-BAPB	0.0061	0.0013	0.99	0.26	3.17	0.95

## Data Availability

The original contributions presented in this study are included in the article. Further inquiries can be directed to the corresponding author.

## Supplementary Materials

The following supporting information can be downloaded at: https://www.mdpi.com/article/10.3390/polym18131595/s1, Table S1: Monomer ratio for preparing different PI Films; Table S2: Df of copolyimide films at different humidity levels (10 GHz, 25 °C); Table S3: Summary of thermal and mechanical properties of 13 types of PI films; Table S4: Dk of copolyimide films at different humidity levels (10 GHz, 25 °C). Figure S1: Infrared spectra of 13 types of PI films; Figure S2: Desorption curves of various PI films saturated with water.
