# Supplementary material for: Achieving Low Dielectric Loss and High Humidity Stability Polyimide Through the Synergistic Effect of Copolymer Monomer Optimization and Aggregation State Regulation
Source: Polymers (Basel). 2026 Jun 26;18(13):1595. doi: 10.3390/polym18131595 (PMC13364279; doi:10.3390/polym18131595)
Supplement: Supplementary file 1 [file polymers-18-01595-s001.zip › polymers-4374166-supplementary.pdf]

Achieving Low Dielectric Loss and High Humidity Stability Polyimide through the Synergistic Effect of Copolymer Monomer Optimization and Aggregation State Regulation

Xing Kang[1], Chenyu Liu[2,*], Hongkui Wu[3], Runxin Bei[1,2], Siwei Liu[1,*] and Yi Zhang[1,*]

[1] PCFM Lab, GD HPPC Lab, Guangdong Engineering Technology Research Center for High-Performance Organic and Polymer Photoelectric Functional Films, GBRCE for Functional Molecular Engineering, State Key Laboratory of Optoelectronic Materials and Technologies, School of Chemistry, IGCME, Sun Yat-sen University, Guangzhou 510275, China

[2] Wuxi ShunXuan Photoelectric Technology CO., LTD, Yixing 214242, China

[3] National Engineering Research Center of Electronic Circuits Base Materials, SHENGYI Technology CO., LTD, Dongguan, Guangdong 523808, China

Table S1. Monomer ratio for preparing different PI Films.

Polyimide	Diamine Ratio（%）				Anhydride Ratio（%）
	TFMB	ODA	aODA	BAPB	BPDA
BPDA-TFMB	100	-	-	-	100
BPDA-85TFMB/15ODA	85	15	-	-	
BPDA-70TFMB/30ODA	70	30	-	-	
BPDA-50TFMB/50ODA	50	50	-	-	
BPDA-ODA	0	100	-	-	
BPDA-85TFMB/15aODA	85	-	15	-	
BPDA-70TFMB/30aODA	70	-	30	-	
BPDA-50TFMB/50aODA	50	-	50	-	
BPDA-aODA	0	-	100	-	
BPDA-85TFMB/15BAPB	85	-	-	15	
BPDA-70TFMB/30BAPB	70	-	-	30	
BPDA-50TFMB/50BAPB	50	-	-	50	
BPDA-BAPB	0	-	-	100	

Table S2. D_f of copolyimide films at different humidity levels (10 GHz, 25°C).

Polyimide	D_f at different RH ($\times 10^{-3}$)								
	**10%**	**20%**	**30%**	**40%**	**50%**	**60%**	**70%**	**80%**	**90%**
TFMB	2.01	2.63	3.29	3.92	4.75	5.63	6.38	6.70	7.80
85TFMB/15BAPB	1.64	2.05	2.28	2.66	3.08	3.59	3.84	4.21	4.90
70TFMB/30BAPB	1.83	2.12	2.51	2.96	3.33	3.85	4.20	4.58	5.01
50TFMB/50BAPB	1.96	2.32	2.69	3.26	3.56	4.04	4.43	4.86	5.28
BAPB	1.99	2.47	3.05	3.62	4.25	5.02	5.61	6.18	6.79
85TFMB/15ODA	1.64	1.99	2.52	3.33	3.94	5.24	5.79	6.44	7.17
70TFMB/30ODA	1.70	1.89	2.34	2.78	3.25	4.54	5.08	5.34	5.92
50TFMB/50ODA	0.88	1.66	2.25	2.86	3.28	4.49	5.01	5.64	6.32
ODA	1.29	1.67	2.39	3.44	4.25	6.17	6.90	8.12	9.11
85TFMB/15aODA	1.64	2.22	2.86	3.42	3.97	4.73	5.20	5.80	6.50
70TFMB/30aODA	1.27	1.76	2.12	2.46	2.80	3.18	3.53	3.96	4.33
50TFMB/50aODA	1.24	1.74	2.23	2.54	3.10	3.40	3.82	4.34	4.77
aODA	1.94	3.15	4.38	5.30	6.47	8.05	9.01	10.14	11.32

Table S3. Summary of thermal and mechanical properties of 13 types of PI films.

Polyimide	T_g[a] (°C)	T_g[b] (°C)	CTE[c] (ppm·K^{-1})	T_d (°C) 1 wt%	T_d (°C) 5 wt%	Tensile Strength (MPa)	Tensile Modulus (GPa)	Elongation at Break (%)
TFMB	350	-	29.3	522	563	233.9±2.2	4.1±0.9	38.6±7.3
85TFMB/15BAPB	268/377	265	37.1	487	545	124.2±2.8	4.0±0.1	10.4±3.6
70TFMB/30BAPB	267/327	263	40.2	486	537	128.3±2.8	3.6±0.1	36.0±9.1
50TFMB/50BAPB	262	262	44.5	477	530	126.4±2.5	3.2±0.5	58.3±7.2
BAPB	287	301	50.9	473	526	144.4±11.5	2.8±0.2	72.3±14.2
85TFMB/15ODA	315	-	33.8	522	566	186.7±5.1	4.2±0.4	48.1±4.5
70TFMB/30ODA	304	265	36.4	514	562	155.0±7.0	4.0±0.1	47.6±4.6
50TFMB/50ODA	277	263	42.3	509	555	142.1±13.0	3.6±0.2	42.3±6.5
ODA	293	293	40.2	494	551	188.4±16.3	3.0±0.5	105.2±18.1
85TFMB/15aODA	316	-	34.2	505	561	189±15.2	4.2±0.2	56.9±18.1
70TFMB/30aODA	272/394	261	45.9	517	561	125.8±9.1	4.0±0.2	9.1±0.9
50TFMB/50aODA	275/371	264	55.2	515	561	122.7±3.9	3.4±0.3	31.4±5.6
aODA	292	289	41.0	467	534	453.1±14.6	3.1±0.5	90.1±23.1

a Measured by DMA, heating rate of 3 °C min^{-1}

b Measured by DSC, heating rate of 10 °C min^{-1}

c Calculated from the second heating curve of TMA (50 to 200 ℃)

Table S4. D_k of copolyimide films at different humidity levels (10 GHz, 25°C).

Polyimide	D_k at different RH								
	10%	20%	30%	40%	50%	60%	70%	80%	90%
TFMB	2.98	3.00	3.02	3.11	3.14	3.16	3.18	3.23	3.27
85TFMB/15BAPB	3.11	3.16	3.22	3.24	3.26	3.25	3.26	3.30	3.35
70TFMB/30BAPB	3.11	3.14	3.17	3.19	3.21	3.22	3.23	3.27	3.31
50TFMB/50BAPB	3.10	3.10	3.10	3.13	3.14	3.17	3.15	3.18	3.20
BAPB	3.18	3.20	3.27	3.29	3.32	3.33	3.35	3.37	3.40
85TFMB/15ODA	2.98	3.06	3.08	3.11	3.13	3.22	3.29	3.30	3.35
70TFMB/30ODA	3.11	3.15	3.17	3.17	3.20	3.21	3.23	3.25	3.28
50TFMB/50ODA	3.17	3.22	3.23	3.22	3.23	3.27	3.29	3.31	3.33
ODA	3.18	3.22	3.24	3.26	3.28	3.33	3.33	3.36	3.38
85TFMB/15aODA	3.02	3.04	3.05	3.07	3.11	3.12	3.11	3.14	3.17
70TFMB/30aODA	3.04	3.10	3.09	3.12	3.13	3.15	3.16	3.18	3.19
50TFMB/50aODA	3.08	3.12	3.11	3.15	3.22	3.20	3.22	3.23	3.25
aODA	3.18	3.19	3.18	3.19	3.24	3.27	3.27	3.29	3.33

Figure S1

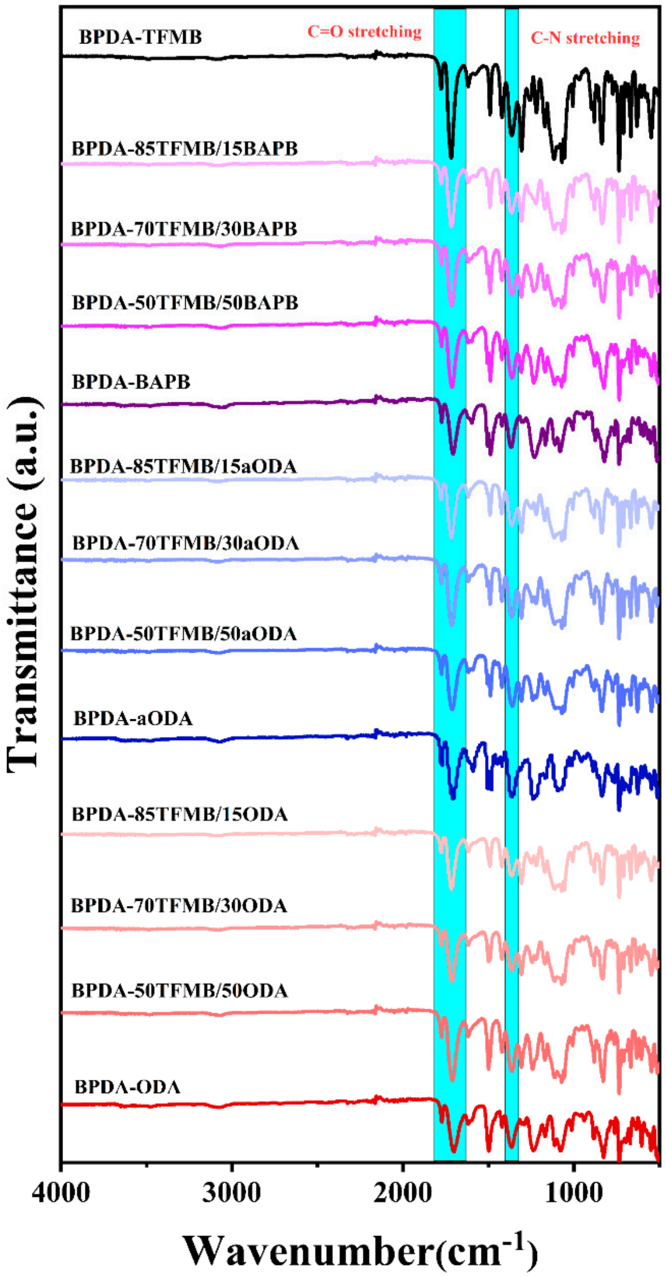

Figure S1. Infrared spectra of 13 types of PI films

Figure S2

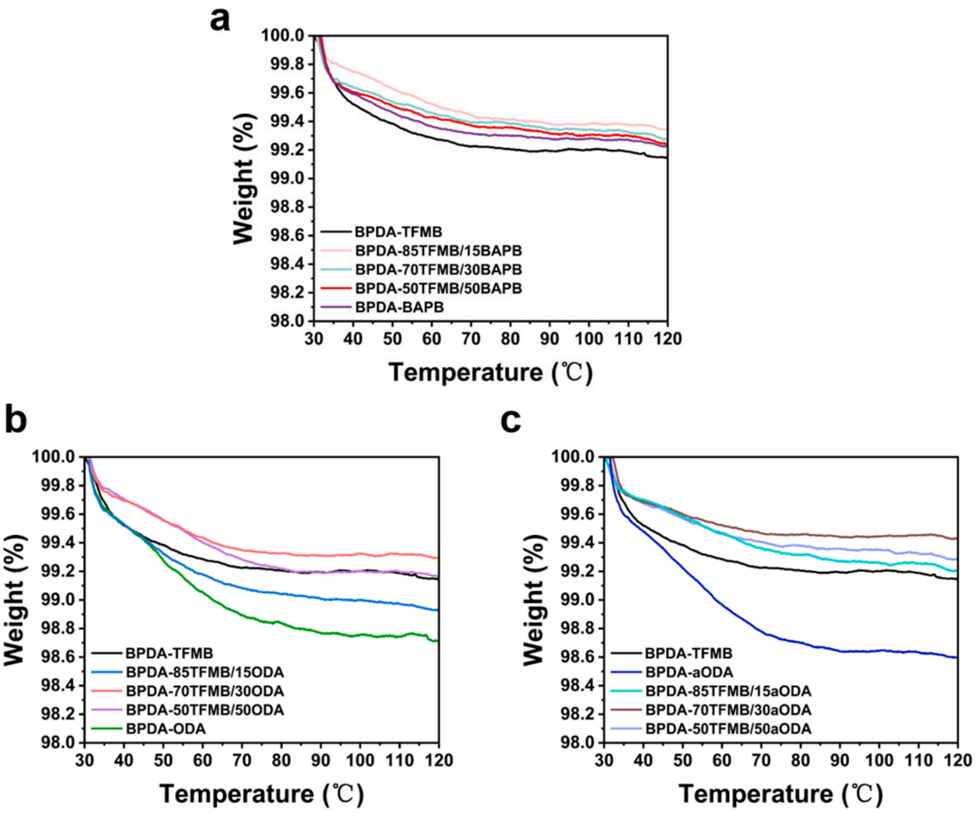

Figure S2. Desorption curves of various PI films saturated with water
